# Networking of Palliative Care at the Corporate Level

**DOI:** 10.4103/0973-1075.76235

**Published:** 2011-01

**Authors:** Kishore Rao, Nagesh Simha

**Affiliations:** Founder Managing Trustee, Karunashraya, Bangalore Hospice Trus, Bangalore, India

**Keywords:** Networking, Palliative Care, Corporate level

## Abstract

This article is a story of networking of palliative care at the corporate level. This gives an insight that if you have will and dedication then you can imagine and make it true that networking can start even before the birth of an organization.

Who would imagine that networking can start even before the birth of an organization? In our case the conception of our (baby) organization had taken place some time before the incident I am going to narrate. The pregnancy was proceeding in a healthy manner. Then sometime in 1991 two persons met while waiting in a queue for coffee during a break in an international conference in Mumbai. One of them was the Medical Director of a hospice in the UK and the other was a person from the Bangalore corporate sector, a dreamer who till then had nothing to do with palliative care. Dr. Jeremy Johnson, had just begun to take an interest in palliative care in India and Kishore Rao, a corporate executive, had begun dreaming of setting up a hospice in Bangalore after his retirement. The occasion was the fifth anniversary of Shanti Avedna Sadan and the participants came from India and many other countries. Shanti Avedna, set up in 1986 was, to my mind, the first formal palliative care centre in the country and the meeting between Dr. Jeremy Johnson and Kishore Rao was quite fortuitous. The meeting was the beginning of a network which has grown into a mature partnership with an exchange of visits definitely once, and sometimes twice, a year.

The Severn Hospice in Shrewsbury, UK is Karunashraya’s twin hospice and there is a free flow of knowledge and expertise in both directions. The networking consists of visits of multidisciplinary teams from Severn and during their visits to Bangalore their doctors, nurses, occupational therapists, physiotherapists, etc. train our staff in the latest developments in palliative care in the UK. Even before the Karunashraya building was ready two of our senior Staff Nurses were trained in Shrewsbury by the Severn staff. There was a time when we, in Bangalore, were concerned that knowledge was flowing only from Shrewsbury to Bangalore and that we had nothing to offer in return. Dr. Jeremy Johnson very graciously put that feeling out of our minds. He explained to us that he and his staff get an enormous sense of fulfillment out of working with a different culture and among our very poor patients. Since we regard Dr. Johnson as our guru maybe what we offer to them is by way of guru dakshina.

It was all very well to dream and plan a hospice, but where were we to get the hands-on carers for the patients. What we obviously needed was a set of trained, experienced nurses who were capable of giving not only physical care but also that element of palliative care that reassures and calms a patient and his family. Shanti Avedna, which had by then become Kishore Rao’s inspiration, was looked after by an order of Nuns called Sisters of the Holy Cross. This organization became his most obvious prospect and he began haunting them to provide nursing care-as and when the Bangalore hospice came up. They were fully extended, they said, and had no spare nurses. Sheer persistence and literally begging their assistance finally paid off and our second network began. At a later very lucky meeting they relented-it appears that their congregation had recently decided to begin looking after the sick and dying and we seem to have been sent to them at the right time! Here was another fortuitous circumstance in the formation of networks. The Sisters came to us to look after, and run, our hospice. Without reservation I can say that the first few years of any organization are the most important for the methods, principles and practices they establish. The Sisters (our second network) firmly established *Karunashraya*, and if it has an excellent reputation for kindness and caring it is because of this network with the Nuns. It is another matter that many years later their priorities changed and our excellent network is now only a happy, grateful, and pleasant memory.

The Indian Cancer Society is one of the parents of the Public Charitable Trust which set up and runs *Karunashraya*. The tremendous task of translating a plan into reality takes much effort and, most importantly, manpower. ICS in Bangalore had only a handful of membersall were sincere and hardworking but this could not make up for sheer numbers. Another chance and fortuitous meeting presented a solution. Two passengers sitting next to each other on a Mumbai-Bangalore flight struck up a conversation-the starting point for our third network. When the subject of the conversation turned to why a corporate executive like Kishore Rao was asking so many searching questions on medical matters to Dr. Nagesh Simha, a practicing Bangalore surgeon, the subject went on to Kishore’s dream of setting up a hospice. Nagesh’s question “What is a hospice” was the beginning of his interest in palliative care. Nagesh is today the Chairman of *Karunashraya* and the President of IAPC and his question also brought his organziation–Rotary Bangalore Indiranagar-as the second parent of the Trust. Our association (networking) with Rotary has brought us dividends not only in manpower, but also in resources. Among the earliest very large donations came from Rotary International which helped to finance the entire equipment bill. Donations have followed at regular intervals. In other matters we have been able to find someone or the other among Rotary members who has “a friend of friend…” and such contacts have helped to get things done.

Bangalore has a Regional Cancer Centre–Kidwai Memorial Institute of Oncology. This is a premier institute for the diagnosis and care of cancer patients. Their patients come from all over the southern states. They provide all-round care and have patients who are really very poor. Because of the work we were doing in the field of cancer the Government of Karnataka had invited Kishore Rao to be on their Board of Visitors and on their Governing Council as an external member. In addition to what he was able to contribute to their running one of the most valuable networks that was formed was our relationship with their institute. We have received every cooperation from them but even more valuable has been their feeding us with patients. They now have a full fledged palliative care department and this has become a major source of patients to receive our care.

Being convinced that palliative care addresses the mental well being of patients and their families, almost as much as does medical care, we had contacted one of the country’s premier institutions–National Institute Mental Health and Neuro Sciences (NIMHANS). This was the start of another healthy network. Today senior members of their faculty train, interact, and guide *Karunashraya’s* staff in all matters concerning counseling and the guiding of our traumatized patients and their families. One more productive and helpful network.

In our early days, we had looked after a young woman with our Home Care team. She passed away after a few weeks and one day soon after we had a visitor in our office who said he was the young ladies’ cousin, that he was very pleased with our compassion and care and “is there something I can do for your young organization”? We were fully expecting a cheque, but he surprised us by asking “Do you have a website”? At that time we did not even own a computer. He said he ran a company which designed websites and offered to make one for us. That was the beginning of our getting on the net or should I say our networking?

There have been other informal networks that have formed in our working lives. The architects of our beautiful, restful, peaceful building designed and supervised the construction entirely free of cost. Our logo, and all the designing of our literature was done, again free of cost by one of the most prominent advertising agencies in south India. There was again no question of payment. Both said that that was their contribution to the cause of the care of the poor and suffering patients. These are early examples of Corporate Social Responsibility and that too these examples came when the now overused term of CSR was only a concept. Incidentally, our architect has received several awards for the design of our building both from India and abroad. Two of our most valuable networks.

We realize we had to struggle to first learn about palliative care, then to collect resources, and end up with the most satisfactory care of almost ten thousand patients and their families. More than five thousand of these have passed away and we can say that almost all of them died in peace and went with dignity. We were able to achieve this with all the networks I have described above. But being a charitable organization we cannot delude ourselves that resources will continue to come to us. Because of this we decided a couple of years ago that we must pass on our expertise and experience to other organizations who wanted to start palliative care services. This was the only way to spread the care of the suffering and also to make palliative care available more widely. Just as we work free for the patients and families we have offered to make our hard earned knowledge available free of charge and with no obligation. Seven smaller palliative care centers have been set up in Chennai, Shimoga, Kalyanpur, Mangalore, Puttur, Vellore, and Nagpur. All the training of their doctors and nursing and counseling staff was done free by us. We also helped them with the knowledge of the planning, equipment identification, purchase, etc. We are now working actively to help set up a hospice in Rishikesh where no palliative care had reached until recently.

Did someone ask how networks start and work? Did someone ask whether palliative care has scope for networking?

